# Nutritional interventions for promoting stress resilience: Recent progress using psychosocial stress models of rodents

**DOI:** 10.1111/asj.13478

**Published:** 2020-11-02

**Authors:** Atsushi Toyoda

**Affiliations:** ^1^ College of Agriculture Ibaraki University Ami Japan; ^2^ United Graduate School of Agricultural Science Tokyo University of Agriculture and Technology Fuchu‐city Tokyo Japan

**Keywords:** depression, mouse, nutrition, resilience, stress

## Abstract

Prevention of stress‐induced adverse effects is important for animals and humans to maintain their quality of life (QOL). Stress decreases the productivity of farm animals and induces abnormal behaviors, which is one of the major problems in animal welfare. In humans, stress increases the risk of mental illness which adversely impacts QOL. Stress is, thus, a common health problem for both animals and humans, and stress prevention and promotion of stress resilience could improve animal and human health and QOL. Among various stresses, psychosocial stress experienced by individuals is particularly difficult to prevent and it could, thus, prove beneficial to attempt to increase resilience to psychosocial stress. There exist a few critical interventions for promoting such resilience, environmental enrichment being one. However, this review describes recent progress in nutritional interventions that could confer resilience to psychosocial stress. The efficacy of this intervention is studied in the social defeat model mouse, which is a standard model for studying psychosocial stress. Several nutrients were found to rescue stress vulnerability using the models. Furthermore, probiotics and prebiotics became crucial dietary interventions for combating psychosocial stress. Collectively, dietary intake of appropriate nutrients will be more important for maintaining QOL in animals and humans.

## 1 INTRODUCTION

Animals, including livestock and humans, are influenced by various environmental stresses. For example, heat stress impacts farm animals and livestock, resulting in a decrease in productivity. In general, several types of stresses which include social stress, adversely affect animals, and, therefore, methods for the reduction in stress need to be implemented for ensure sustained animal production. In humans, psychosocial stress is one of the most serious problems affecting human health and society, and have been shown to increase the risk of mental illness, such as depression and anxiety (American Psychiatric Association, 2013). Proper intervention is essential to prevent suicide, one of the fatal outcomes induced by mental illness (Thornicroft & Sartorius 1993). There are several critical interventions for combating stress‐induced disorders, especially some food and feed ingredients such as phytochemicals have potential benefits for conferring stress resilience. For example, the food substances with the activities of anti‐inflammation are focused on because central and peripheral inflammations are linked to psychiatric disorders (Hodes et al., 2014).

Actually, stressful events do not always induce significant abnormalities, such as behavioral deficits and suppressed growth, in all animals. Furthermore, in humans, psychosocial stress induces depression in people vulnerable to stress, and the majority who show resilience to stress can adapt to stress and maintain their psychological well‐being. Collectively, dietary intervention with proper substances will give significant benefits for stress vulnerable individuals.

In this review, the progress in recent basic research undertaken to study resilience and the nutritional factors that contribute to the promotion of resilience will be described. Finally, the application of these findings to animal science will be discussed.

## 2 ANIMAL MODELS FOR RESILIENCE RESEARCH

The most advanced knowledge on resilience to psychosocial stress is from research studies that use socially defeated rodents, which represents one of the main depression models (Cathomas et al., 2019; Der‐Avakian et al., 2014; Miczek, 1979; Russo et al., 2012). The methods for producing the social defeat model mice are described in detail in literature (Golden & Covington, 2011; Goto & Toyoda, 2015). It is based on the resident‐intruder paradigm, in which the intruder male mouse is socially defeated by the resident and aggressive male mouse that is in the home cage. Normally, C57BL/6 (B6) or BALB/c (BALB) mice are preferred for use as the intruders, namely, socially defeated models, and ICR (CD1) mice are preferred for use as the aggressor residents. In this model, ICR would attack and defeat B6 socially. Sensitivity to psychosocial stress, resilience, and susceptibility are measured mainly by the social interaction (SI) test, which reveals social interaction between the resident and the intruder in the open field box (Goto & Toyoda, 2015). Intruder mice that show social avoidance with ICR mice are susceptible, while intruder mice that show social interaction with ICR are resilient. Previous studies have reported that B6 mice are more resilient to social defeat stress compared to BALB (Razzoli et al., 2011; Savignac et al., 2011; Yamagishi et al., 2019). There are some potential variations in the methods involved in producing mice for the social defeat model. Furthermore, the vicarious social defeat stress paradigm especially consists only of psychological stress, and not physical stress experienced by the residents (Sial et al., 2016), and produces model animals with high face, construct, and predictive validity for depressive disorders. Unfortunately, the social defeat model was limited, in that the previous trials failed to develop female models of social defeat. Recently, however, the socially defeated female models had been successfully developed by several unique techniques, including the vicarious social defeat stress paradigm (Harris et al., 2018; Iñiguez et al., 2018; Newman et al., 2019; Takahashi et al., 2017). These rodent models of social defeat have been mainly used to reveal the mechanisms of mental illness, such as depression and post‐traumatic stress disorder; as such, hopefully these models could be used for basic and applied research on livestock and zoo animals because group‐housed livestock and zoo animals are constantly subjected to social defeat stress (Toyoda, 2017). Recently, it was discovered that non‐purified feed increases stress resilience in mice subjected to subchronic and mild social defeat stress (sCSDS) compared to semi‐purified feed (Goto et al., 2016). These observations related to animal feed and nutrition in model mice may contribute to improve the livestock production.

## 3 MECHANISMS OF RESILIENCE

Wide varieties of studies on resilience mechanisms are carried out using social defeat models of mice and rats. Krishnan et al. (2007) discovered that inbred B6 mice subjected to chronic social defeat stress (CSDS) were separated into susceptible and resilient subpopulations. The susceptible mice showed several behavioral deficits, such as social avoidance, however, these deficits were absent in the resilient mice. The susceptible and the resilient mice have, therefore, been compared in terms of many aspects (Cathomas et al., 2019; Koo et al., 2019). The rewards system in the brain has been the main focus of these experiments, and the projection of dopamine (DA) neurons in the ventral tegmental area (VTA) to the nucleus accumbens (NAc) has been well characterized using electrophysiological and molecular approaches (Russo et al., 2012). Furthermore, the prefrontal cortex and hippocampus have been analyzed for the elucidation of resilience mechanisms (Anacker et al., 2018; Corbett et al., 2019). Recently, the immune system, blood brain barrier (BBB), intestinal microbiome, etc., have become the potential targets for resilience research, and the relationship between chronic inflammation and stress vulnerability has been well studied (Cathomas et al., 2019). Hereafter, each of these areas of research focus will be discussed.

### 3.1 VTA and NAc

DA projection of VTA toward NAc is known as a reward pathway in the brain and its physiological deficit is involved in drug addiction and depression (Russo et al., 2012). As described above, Krishnan et al. (2007) utilized a large cohort of B6 mice exposed to CSDS, and successfully discovered that CSDS induces two subpopulations that have behavioral characteristics that could characterize them as mice who are vulnerable and mice who are resilient to social stress. The difference in sensitivity to psychosocial stress in B6 mice is based on the electrophysiological properties of VTA DA neurons projecting to NAc. Higher frequency of spikes of DA neurons from VTA to NAc was observed in susceptible mice compared to resilient mice. In addition, the comprehensive analyses for gene expressions in VTA and NAc revealed that gene expression patterns differ between resilient and susceptible mice, and several critical genes for stress vulnerability were identified and characterized (Christoffel et al., 2011; Dias et al., 2014; Golden et al., 2013). Furthermore, there is a significant morphological difference in the medium spiny neurons (MSNs) in NAc of resilient and susceptible mice, and both D1 and D2 MSNs in susceptible mice showed more extensive dendrites (Cathomas et al., 2019). Although, neuronal tissues have been the major targets of studies related to stress resilience, other tissues such as blood vessels in the brain serve as more attractive targets for these studies. Recently, permeability of BBB in NAc was found to be critical for the determination of stress sensitivity (Menard et al., 2017). Claudin 5 (Cldn5) is a major protein of endothelial cell tight junctions in BBB (Morita et al., 1999; Nitta et al., 2003), and recently Menard et al. (2017) discovered that Cldn5 of NAc is an important molecule for determination of stress susceptibility and resilience. In stress‐susceptible B6 mice, low‐level expression of Cldn5 and abnormal tight junctions in NAc were observed that reduced BBB integrity, following infiltration of peripheral cytokine interleukin‐6 (IL‐6) into the brain parenchyma, and leading to subsequent expression of depression‐like behaviors.

### 3.2 Prefrontal cortex (PFC)

The PFC is affected by various stresses, including psychosocial stress and PFC damage, such as synapse loss, dendrite atrophy, and reduced myelination, has been observed in depressive patients and the depression animal models. Therefore, this region is well‐studied to elucidate mechanisms of stress resilience (Kang et al., 2012; Lehmann et al., 2017; McEwen & Morrison, 2013; Palazidou, 2012). The VTA‐medial prefrontal cortex (mPFC) projection of DA neurons plays a critical role in stress resilience of CSDS mice (Chaudhury et al., 2013). In the social defeat model mouse, PFC mRNA expression of corticotrophin‐releasing factor was increased in populations of susceptible mice compared to that of resilient mice (Gururajan et al., 2019). Studies on resilience mechanisms were especially focused on the mPFC, which plays a significant role in working memory, decision‐making, and higher‐level cognitive functions. Interestingly, a recent study described that low‐level expression of micro‐RNA miR‐218 in mPFC increases stress susceptibility, whereas the overexpression of miR‐218 selectively in mPFC pyramidal neurons promotes resilience to CSDS (Torres‐Berrio et al., 2019). Torres‐Berrio et al. (2017) described that miR‐218 is a posttranscriptional repressor for the Netrin‐1 guidance cue receptor *DCC* (deleted in colorectal cancer) and was expressed in pyramidal neurons of PFC in both humans and mice. Reduced miR‐218 and overexpressed *DCC* in the PFC are also observed in stress‐susceptible mice and patients with major depressive disorder. Another study revealed that the interaction between the cyclic monophosphate (AMP) response element binding protein and the zinc finger protein (Zfp189) in the PFC plays a potential role in stress resilience (Lorsch et al., 2019). A metabolomics approach was used to elucidate the resilience features of the brain in the rodent model, and it revealed higher levels of inosinic acid and adenosine AMP in mPFC in resilient mice compared to susceptible mice (Dulka et al., 2017).

### 3.3 Immune system

Psychosocial stresses impact the innate and adaptive immune systems and induce mental disorders (Dantzer, 2018). Several key factors in the inflammatory reactions are involved in depressive disorder and stress resilience in CSDS mice (Cathomas et al., 2019). Peripheral leukocyte populations and interleukin‐6 (IL‐6) levels have been shown to positively correlate with social avoidance, namely, stress susceptibility in CSDS mice (Hodes et al., 2014). Furthermore, CSDS‐induced peripheral inflammatory factors, including IL‐6 enter the NAc through leaky BBB in the stress susceptible mice, as described above (Menard et al., 2017). In the central nervous system, CSDS increases the expression of chemokine (C‐C motif) ligand 2 in microglia, which induces brain infiltration of monocytes and inflammation, following a deficit of social behavior (Furuyashiki & Kitaoka, 2019). Moreover, decreased toll‐like receptor 2/4 (TLR2/4) in mPFC failed to induce stress susceptibility in CSDS mice. Therefore, TLR2/4 in mPFC may play a critical role in the regulation of sensitivity for psychological stress (Nie et al., 2018).

### 3.4 Gut microbiota

The risks of several diseases link to the microbiota‐gut‐brain axis, which also plays a critical role in stress‐induced disorders, such as depression (Foster et al., 2017). A previous study showed that CSDS changed the gut microbiota in male mice, and several bacterial abundances in both phyla and genera correlated with stress‐resilience (Szyszkowicz et al., 2017). These microbial changes in the most susceptible mice correlated with the expression of interleukin 1β and IL‐6 mRNA in PFC. Another study showed that *Bifidobacterium* in feces was detected only in resilient mice of the CSDS model, but not in susceptible mice (Yang et al., 2017). Surprisingly, this study also showed that oral intake of *Bifidobacterium* induces stress‐resilience in CSDS mice. Interventions of probiotics and prebiotics for social defeat models were frequently carried out to change the gut ecosystem, which will be described in the part below.

## 4 RESILIENCE AND NUTRITION

Dietary habit is considered to influence mental and brain conditions. It has been found that diet quality impacts stress sensitivity in sCSDS mice (Goto et al., 2016, 2017). This result conveys the possibility that the ingredients of feeds and foods may promote stress resilience. Here, the recent progress in research studying the relation between stress resilience and nutrition using a mouse model of social defeat is introduced. Table 1 shows that food‐related substances with significant functions increase resilience to stress in the social defeat model of mice. Below, several promising nutritional factors that could be used to increase stress resilience are discussed.

**TABLE 1:** Food‐related substances increasing resilience to stress

Substance	Stress model	Application	References
Phytochemicals			
Hesperidine	sCSDS	Feed additive	Sato, Okuno, et al. (2019)
Sulforaphane	CSDS	i.p.	Yao et al. (2016)
Glucoraphanin	CSDS	Feed additive	Yao et al. (2016)
Bioactive dietary polyphenol preparation	CSDS	Water additive	Wang et al. (2018)
Dihydrocaffeic acid and malvidin−3‐*O*‐glucoside	CSDS	Water additive	Wang et al. (2018), Blaze et al. (2018)
Curcumin	CSDS	Feed additive	Aubry et al. (2019)
Caffeine	CSDS	i.p.	Yin et al. (2015)
Amino acids, peptides,A31 and others			
Branched‐chain amino acids	CSDS	i.p.	Nasrallah et al. (2019)
Leucine–histidine dipeptide	CSDS	p.o.	Ano et al. (2019)
Lactate	CSDS	i.p.	Karnib et al. (2019)
Probiotics			
*Lactobacillus helveticus* strain MCC1848	sCSDS	Feed additive	Maehata et al. (2019)
*Bifidobacterium* ^a^	CSDS	p.o.	Yang et al. (2017)
Prebiotics			
FOS + GOS^b^	CSUS^c^	Water additive	Burokas et al. (2017)

^a^ LAC‐B Granular Powder, Kowa Pharmaceutical, Ltd, Tokyo, Japan.

^b^ Fructo‐oligosaccharide and galacto‐oligosaccharide.

^c^ Chronic social unpredictable stress.

### 4.1 Phytochemicals

Several phytochemicals have the potential to help increase resilience to psychosocial stress. Sato, Okuno, et al. (2019) discovered that the peel of immature local citrus, *Citrus tumida* Hort. Ex Tanaka (*C. tumida*), shows a slight activity that increases stress resilience, namely, decreased social aversion in the SI test using a sCSDS model mouse. Social avoidance in the model mouse is linked to inflammation in the brain, as described above; hence, hesperidin, the constituent of the citrus peel showing an anti‐inflammatory activity, was studied. Surprisingly, a semi‐purified diet containing 0.1% hesperidin significantly rescued social avoidance in sCSDS mice and increased levels of kynurenine, which is one of the major metabolites involved in inflammation in the hippocampus and the PFC (Sato, Okuno, et al., 2019). In addition, this peel has an anti‐obesity effect on mice that are fed a high‐fat diet (Sato et al., 2019). It can, thus, be proposed that *C. tumida* could serve as a beneficial food item in the prevention of lifestyle‐related diseases, including depression.

Also, sulforaphane in broccoli sprouts, and its precursor, glucoraphanin, has been shown to increase the stress resilience of CSDS mice (Yao et al., 2016). Pre‐feeding of unpurified diet containing 0.1% glucoraphanin for 11 days prior to CSDS exposure made the CSDS mice resilient in the SI test, and rescued anhedonia in the sucrose preference test. These compounds present in broccoli sprouts could increase stress resilience in CSDS mice through the Kelch‐like ECH‐associated protein 1‐NRF2 system, which reduces the cell damage by reactive oxygen species (Yao et al., 2016).

Furthermore, bioactive dietary polyphenol preparation (BDPP) derived from grape seeds shows a function that helps to increase stress resilience (Wang et al., 2018). This study described two phytochemicals, dihydrocaffeic acid (DHCA) and malvidin‐3′‐*O*‐glucoside (Mal‐gluc) in BDPP, that were identified as potential ingredients that contribute to the resilience of B6 mice. Wang et al. (2018) showed that DHCA decreases expression of IL‐6, while Mal‐glu increases small GTPase Rac1 expression via epigenetic modifications. Both IL‐6 and Rac1 are implicated with stress vulnerability in mice and humans (Golden et al., 2013; Hodes et al., 2014). DHCA and Mal‐gluc treatments changed stress‐induced DNA methylation in the peripheral blood mononuclear cells of the hippocampus (Blaze et al., 2018). In addition, other groups reported that caffeine and curcumin confer stress resilience in CSDS mice (Aubry et al., 2019; Yin et al., 2015).

In summary, recent studies have shown various phytochemicals to possess biological functions that help in the development of stress resilience. The anti‐inflammatory actions of these phytochemicals may play a key role in fighting psychosocial stress. Since food and feed substances have various phytochemicals with anti‐inflammatory activities, the screening of these compounds using social defeat models will enable the discovery of molecules that confer resilience more effectively (Zhang et al., 2019).

### 4.2 Amino acids, peptides, and others

Some amino acids and peptides have been shown to confer resilience to psychosocial stress. Branched‐chain amino acids (BCAA) promote stress resilience and rescue social avoidance via activation of hippocampal brain‐derived neurotrophic factor signaling (Nasrallah et al., 2019). Moreover, leucine–histidine (LH) dipeptide promotes stress resilience in CSDS mice through inhibition of CSDS‐induced microglial activation (Ano et al., 2019). Exercise has significant benefits for health in animals and humans, and can possibly relieve mental illness, such as depression (Kim & Leem, 2016; Mul et al., 2018). Blood levels of lactic acid are elevated by extensive exercise, which is considered to be linked to fatigue. Surprisingly, a recent study revealed that lactic acid modifies hippocampal histone deacecylases (HDAC2/3) and promotes stress resilience in mice (Karnib et al., 2019).

### 4.3 Probiotics

As mentioned above, the brain‐gut‐microbiota axis is a promising and critical target in basic and clinical research on mental disorders. Probiotics represent a major intervention for the recovery of the gut ecosystem, especially *Lactobacillus* and *Bifidobacterium*, which are potent probiotics that promote resilience to psychosocial stress. Several studies have reported that probiotic intervention confers stress resilience in social defeat models. Maehata et al. (2019) reported that dietary intervention of the heat‐killed *Lactobacillus helveticus* strain MCC1848 (MCC1848) confers resilience in the sCSDS model mice. Furthermore, MCC1848 modulates the NAc gene expression patterns in stress‐susceptible mice, which may be critical in its MCC1848 function. Also, an another report described that oral administration of *Bifidobacterium* increased stress resilience in CSDS mice (Yang et al., 2017).

### 4.4 Prebiotics

Prebiotics is another intervention used, apart from probiotics, to change gut microbiota that may in turn, prove beneficial in developing resilience to psychosocial stress. Burokas et al. (2017) found that the combination of fructo‐oligosaccharides (FOS) and galacto‐oligosaccharides (GOS) improves stress resilience in mice subjected to chronic social unpredictable stress, a modified version of CSDS consisting of both unpredictable exposure of social defeat and overcrowding stress. This study also showed that the combined application of FOS and GOS suppresses forced swimming stress‐induced hyperthermia and corticosterone elevation compared with individual interventions of FOS and GOS.

## 5 PERSPECTIVES AND CONCLUSION

Appropriate feeds are important to enable livestock to efficiently produce animal products and maintain QOL. This review introduced and discussed recent progress in the discovery of food‐related substances (Table 1) that are capable of conferring resilience to psychosocial stress in the mouse models of social defeat, which is a standard model used in studies pertaining to depressive disorders. These substances, with their capacity for promoting resilience, could contribute to the development of novel functional feeds for livestock and zoo animals that could help prevent psychosocial stress‐induced deficits in these animals.

## CONFLICT OF INTEREST

The author was supported by the research grants from Morinaga Milk Industry Co., Ltd.
